# Metabolite aberrations in early diabetes detected in rat kidney using mass spectrometry imaging

**DOI:** 10.1007/s00216-019-01721-5

**Published:** 2019-03-20

**Authors:** Hilde-Marléne Bergman, Lina Lindfors, Fredrik Palm, Jan Kihlberg, Ingela Lanekoff

**Affiliations:** 1 Department of Chemistry-BMC, Uppsala University, Box 599, 751 24 Uppsala, Sweden; 2 Department of Medical Cell Biology, Uppsala University, Box 571, 751 23 Uppsala, Sweden

**Keywords:** nano-DESI, Acylcarnitine, Fatty acid oxidation, Branched-chain amino acid, Streptozotocin, Type I diabetes

## Abstract

Diabetic kidney disease is a serious complication of diabetes that can ultimately lead to end-stage renal disease. The pathogenesis of diabetic kidney disease is complex, and fundamental research is still required to provide a better understanding of the driving forces behind it. We report regional metabolic aberrations from an untargeted mass spectrometry imaging study of kidney tissue using an insulinopenic rat model of diabetes. Diabetes was induced by intravenous injection of streptozotocin, and kidneys were harvested 2 weeks thereafter. Imaging was performed using nanospray desorption electrospray ionization connected to a high-mass-resolving mass spectrometer. No histopathological changes were observed in the kidney sections; however, mass spectrometry imaging revealed a significant increase in several 18-carbon unsaturated non-esterified fatty acid species and monoacylglycerols. Notably, these 18-carbon acyl chains were also constituents of several increased diacylglycerol species. In addition, a number of short- and long-chain acylcarnitines were found to be accumulated while several amino acids were depleted. This study presents unique regional metabolic data indicating a dysregulated energy metabolism in renal mitochondria as an early response to streptozotocin-induced type I diabetes.

**Electronic supplementary material:**

The online version of this article (10.1007/s00216-019-01721-5) contains supplementary material, which is available to authorized users.

**Figure:**
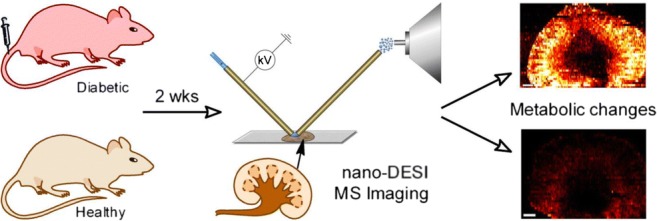
Graphical abstract

## Introduction

Diabetic kidney disease (DKD) is a common complication of diabetes that can lead to end-stage renal disease (ESRD). ESRD is a fatal condition requiring dialysis and ultimately renal transplantation [1]. Despite decades of research, the underlying molecular mechanisms behind DKD are still debated and not well understood [2, 3]. One approach to understanding disease pathophysiology is to study the chemical alterations resulting from the diseased state. An increasingly popular group of molecules to study for understanding pathophysiology is small metabolites. Small metabolites are found as intermediate and final products of all chemical processes in living systems and metabolic profiling of biological samples has become a viable approach due to recent technological advancements [4].

Metabolic profiling provides detailed analysis of small metabolites in a sample and can be used to understand altered metabolic pathways in disease states. Mass spectrometry (MS) is a common technique for metabolic profiling for both targeted and untargeted analyses [5]. One main advantage of using MS for metabolomics lies in its untargeted nature and ability to detect hundreds of chemical species simultaneously without labelling or preselection. The features of MS have been important in studies screening biofluids—for metabolites as biomarkers for diagnosis of DKD, as well as to predict progression to ESRD [6].

In addition to analysing biofluids, MS can be used to acquire data directly from defined locations on the surface of thin tissue sections using mass spectrometry imaging (MSI) [7, 8]. By collecting spatially resolved MS data, the distribution and relative abundance of detected ions can be visualised in two-dimensional (2D) maps. These 2D maps provide unique insights into chemical microenvironments at selected locations and thereby the biochemical system. Previous MSI studies on kidneys of diabetic rodent models indicate the importance of lipids and energy metabolism in disease pathology [9, 10]. Specifically, the kidney of db/db mice with DKD 16 weeks after diabetes onset showed anatomically specific increases of four lipid classes: gangliosides, sulfoglycosphingolipids, lysophospholipids, and phosphatidylethanolamines [9]. In addition, the level of sphingomyelin 18:1/16:0 and the ATP/AMP ratio were increased in the glomeruli of Akita mice with DKD 19 weeks after diabetes onset [10]. These studies show that MSI can be used to register metabolic alterations in DKD > 16 week after diabetes onset and advance knowledge on disease progression.

In the present study, we employ nanospray desorption electrospray ionization (nano-DESI) MSI to investigate metabolic alterations in thin kidney tissue sections from the insulinopenic streptozotocin (STZ) type 1 diabetes rat model, 2 weeks after disease onset [11, 12]. nano-DESI is a liquid extraction technique where analytes are desorbed from the sample surface in ambient conditions and with minimal sample preparation. For imaging, the sample is continuously moved under the nano-DESI probe while data is continuously acquired. This generates a matrix of mass spectra where each spectrum corresponds to a specific point on the sample surface. While previous metabolomic studies focused on investigating chemical changes in tissue of diabetic kidney or DKD after > 16 weeks [13, 14], this study focuses on metabolic alterations that occur only 2 weeks after disease onset. We report that despite the lack of histological alterations at this early stage, the metabolism has already shifted. In particular, there is a dysregulation of the citric acid cycle, fatty acid oxidation (FAO), and branched-chain amino acid (BCAA) catabolism within the renal cortex. These results provide new insights into the complex metabolic consequences of diabetes and disease progression.

## Materials and methods

### Animals and sample preparation

All animal procedures were approved by the local animal ethics committee in Uppsala and performed in accordance with the National Institutes of Health Guide for the Care and Use of Laboratory Animals. Diabetes mellitus was induced by injection of STZ (55 mg/kg bw, Sigma-Aldrich, St. Louis, MO, USA) in the tail vein of male Sprague-Dawley rats (Charles River, Sulzfeld, Germany) weighing approximately 300 g [15]. Blood glucose concentrations were determined 24 h after injection using a test reagent strip from blood samples obtained from a cut of the tip of the tail. Animals were considered diabetic if blood glucose concentrations increased to ≥ 15 mmol/l. Age-matched normoglycaemic rats were used as controls. Two weeks after induction of diabetes, animals were anaesthetised (*n* = 3/group) with thiobutabarbital (i.p., 120 and 80 mg/kg bw for controls and diabetic animals, respectively, due to their different responses to anaesthetics), and kidneys were rapidly dissected and frozen in liquid nitrogen. One kidney per animal was sectioned into 12-μm-thick sections (Leica CM3000, Leica Biosystems, Nussloch, Germany) and thaw mounted onto regular glass slides. Water was used to hold the fresh frozen tissue during sectioning, and all sections were stored at − 80 °C prior to nano-DESI MSI analysis.

Kidney function (data presented in the Electronic Supplementary Material (ESM) Table S1) was determined in parallel in both control and diabetic animals (*n* = 4/group) as previously described [16]. In brief, anaesthesia was induced using thiobutabarbital and body temperature maintained at 37.5 °C using a servo-controlled heating pad. Tracheostomy was done, and catheters were inserted into the femoral artery for monitoring of the mean arterial blood pressure, into the femoral vein for infusion of 3H-inulin (185 kBq h^−1^ kg^−1^; controls 5 ml h^−1^ kg^−1^ and diabetics 10 ml h^−1^ kg^−1^), and into the bladder to allow drainage. The left kidney was immobilised in a plastic cup and the left ureter was catheterised in order to collect urine for subsequent measurements of left kidney function. Glomerular filtration rate was calculated from the urinary excretion rate of 3H-inulin, using a standard liquid scintillation technique [17]. Urinary protein concentration was analysed according to manufacturer’s protocol (DC protein assay, Bio-Rad Laboratories, Sundbyberg, Sweden).

### nano-DESI MSI

A custom-built nano-DESI MSI source was assembled as previously described [11], and a capillary holder was used to fix the primary and secondary capillaries at an angle [18]. The nano-DESI solvent consisted of a 9:1 solution of methanol:water (*v*/*v*), with an addition of 3 μmol/l lysophosphatidylcholine 19:0, and was supplied at a rate of 0.5 μl/min. Imaging was performed by moving the sample in lines under the nano-DESI probe at a speed of 60 μm/s in the *x*-direction and a step size of 200 μm between the lines in the *y*-direction. The average acquisition time of 0.7 s per spectrum resulted in pixel sizes of ~ 42 × 200 μm. Three tissue sections from one kidney each of 3 control and 3 STZ-treated rats were analysed by nano-DESI MSI in a random order.

All mass spectrometric analyses were performed in an untargeted fashion on a Q-Exactive™ Plus Orbitrap™ (Thermo Fisher Scientific, Bremen, Germany). MSI data acquisition was performed in positive mode with a scan window of *m/z* 100–1000, using a mass resolution of 140,000 (*m/*Δ*m* at *m/z* 200). The instrument was externally calibrated, the spray voltage was set to 3 kV, and the heated capillary temperature was set to 300 °C.

### Data analysis

After nano-DESI MSI, the analysed tissue sections were stained by haematoxylin & eosin (H&E). The protocol is described in the ESM. Regions of interest (ROIs) of the cortex together with the outer strip of the outer medulla, and the inner strip of the outer medulla together with the inner medulla, were manually defined based on optical images of the stained tissue sections. Microscopy images of H&E-stained tissue sections were used for histological evaluation.

Data containing *m/z* values and intensities were extracted from Xcalibur raw files using Decon2LS [19]. Following this, data matrices were generated and mass spectra were extracted from defined ROIs using an in-house script [20]. For further comparisons, all intensities were normalised to the total ion current (TIC) and increased intensities were interpreted as increased abundances. Welch’s *t* test was used to select *m/z* values with significantly (*p* < 0.05) different relative intensities between control and diabetic tissues. Only *m/z* values that were present in > 5% of the pixels in each ROI and in > 25% of all tissue sections were chosen for further investigation. In addition, only *m/z* values showing significant differences in both the [M+Na]^+^ and [M+K]^+^ ion channels were selected. All abundances are interpreted from TIC-normalised data. Ion images were generated using MSIQuickView, and the localisation of all biologically relevant peaks to the kidney tissue was verified by manual inspection [11].

### Analyte identification

The total number of endogenous compounds detected in a control tissue section was estimated by searching all detected *m/z* values in the human metabolome database (http://www.hmdb.ca) and Metlin (https://metlin.scripps.edu) to exclude biologically irrelevant peaks. The number of detected endogenous compounds was determined by removing all duplicate hits with the same elemental composition in addition to ions detected as several adduct ions. Analyte identification strategies are further described in the ESM.

## Results

**Fig. 1:**
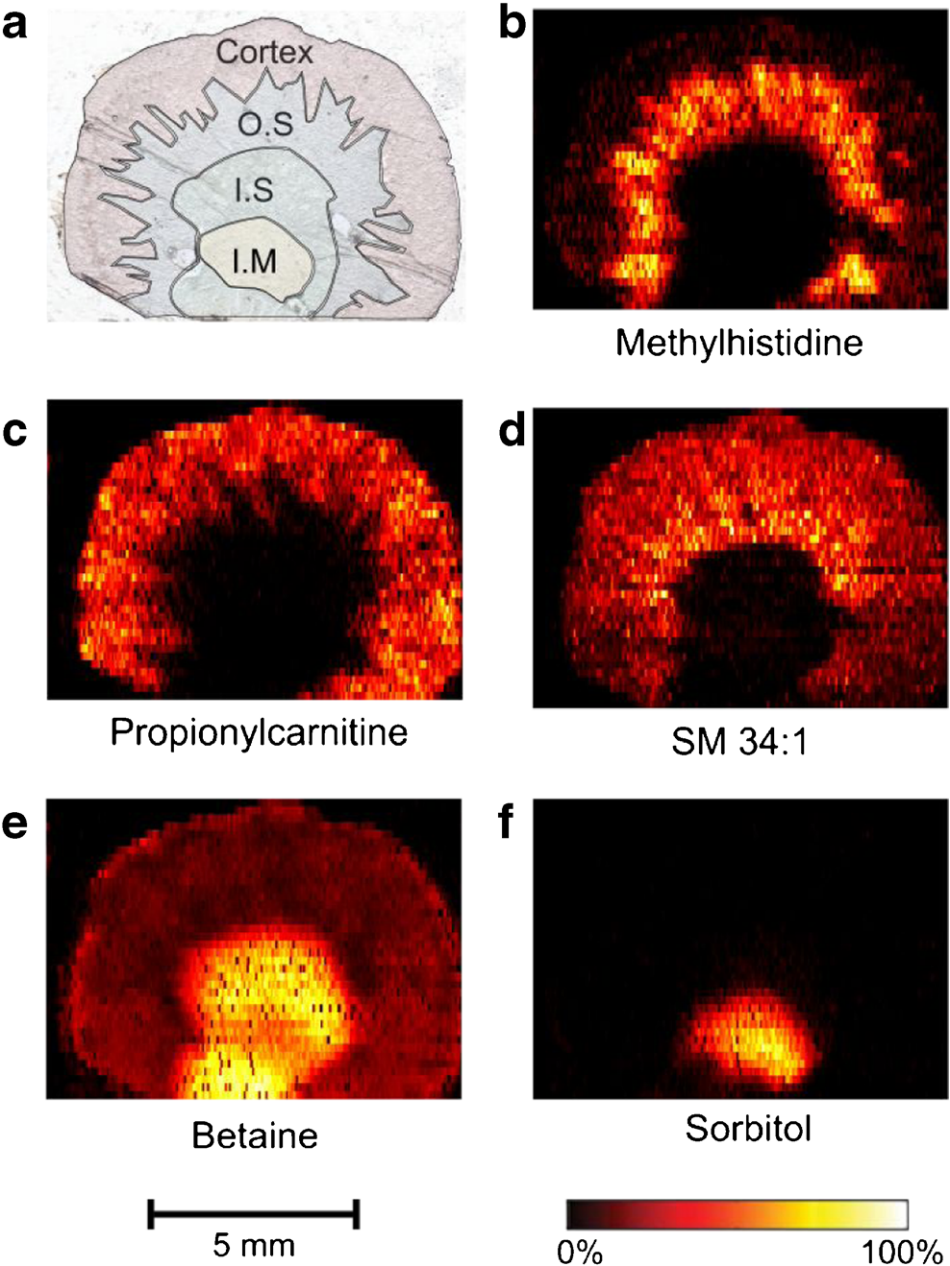
Anatomical regions of kidney tissue have distinct molecular composition. (**a**) Optical image of kidney section with an overlay highlighting anatomical regions. (**b**) Ion image of [methylhistidine+H]^+^ (*m/z* 170.0923). (**c**) Ion image of [propionylcarnitine+H]^+^(*m/z* 218.1386). (**d**) Ion image of [sphingomyelin 34:1+K]^+^ (*m/z* 741.5307). (**e**) Ion image of [betaine+Na]^+^ (*m/z* 140.0681. (**f**) Ion image of [sorbitol+Na]^+^ (*m/z* 205.0681)

Kidney tissue contains distinct anatomical regions responsible for activities such as filtration of blood and formation of urine. Figure 1a highlights the four major anatomical regions in a transverse kidney section: cortex, outer strip of the outer medulla (OS), inner strip of the outer medulla (IS), and inner medulla (IM). Ion images generated with nano-DESI MSI reflect these anatomical regions and reveal their differences in chemical composition. More than 250 ion images of low molecular weight ions with unique chemical formulas were acquired from kidney tissue sections with nano-DESI MSI. Of all the detected ions, the majority localise to the OS and/or the cortex, while 50 ions are distributed evenly over the tissue section and ~ 75 ions are localised to the IS. Methylhistidine (Fig. 1b) is, for example, more abundant in the OS and propionylcarnitine (C3) is mainly localised to the cortex (Fig. 1c). While these metabolites show complementary distributions, the membrane lipid sphingomyelin 34:1 localises to both of these regions (Fig. 1d). Further, betaine [21] (Fig. 1e) is mainly localised to the IS and sorbitol (Fig. 1f) mainly to the IM. The large amount of metabolites detected and imaged with nano-DESI MSI can provide novel insights into localised metabolism and biological function in kidney tissue.

### Metabolism is altered in the diabetic kidney tissue

**Fig. 2:**
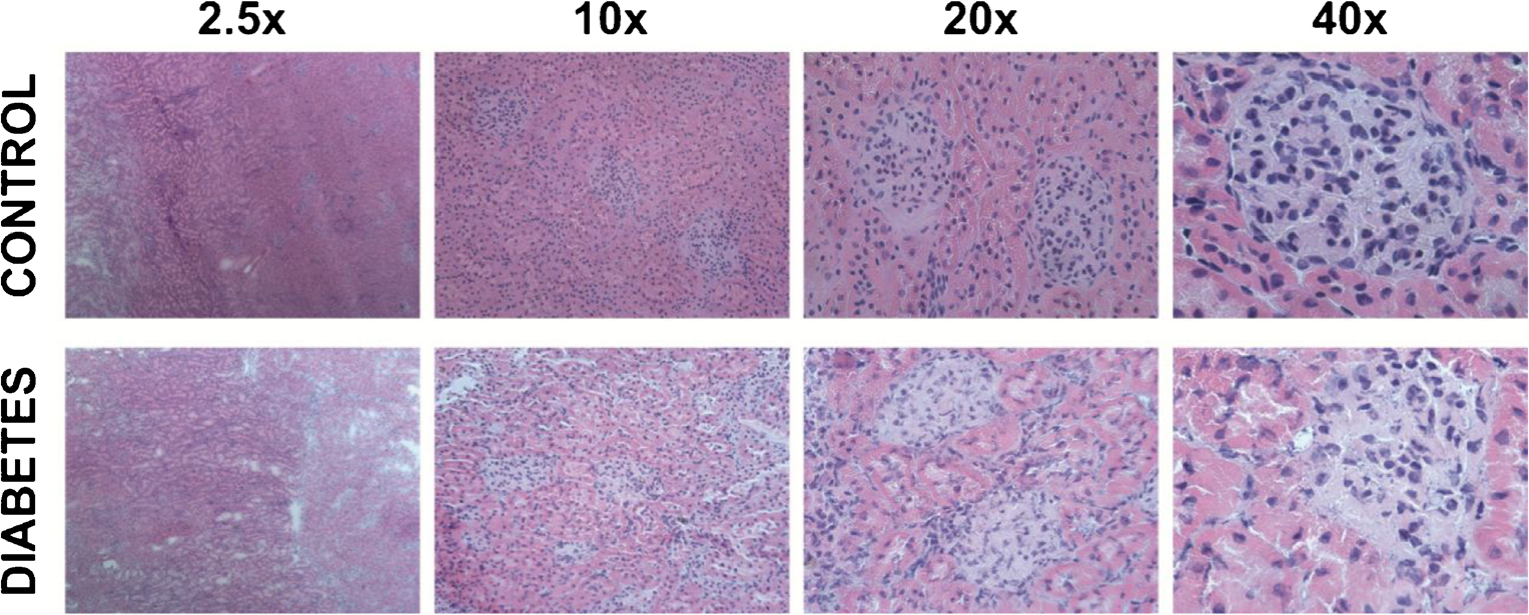
Representative optical images of H&E-stained control and diabetic kidney sections, 2 weeks post STZ treatment, at four different magnifications

Two weeks after STZ treatment, rats were deemed diabetic with high glucose blood levels. In addition, they showed signs of kidney dysfunction, such as increased glomerular filtration rate, urine flow, and urinary protein excretion (ESM Table S1). However, at this early stage of disease, there were no pathological changes correlating to DKD observed in the cortex during histological evaluation of the tissue (Fig. 2). The lack of histological changes in the tissue, despite clear signs of kidney dysfunction, suggests that metabolic alterations are involved in early disease pathogenesis. Metabolite distributions and abundances in thin kidney tissue sections were imaged by nano-DESI MSI using three sections from one kidney each of 3 age-matched controls and 3 STZ diabetes model rats, 2 weeks after STZ treatment. The results show a significant change in abundance of 38 annotated endogenous metabolites, including glucose, in the cortex and OS of the kidney (Table 1, details on annotation in ESM Tables S2-S4 and S6-S11). Specifically, the abundance of 24 metabolites was significantly increased while the abundance of 14 metabolites, including several amino acids, was significantly decreased (*p* value < 0.05, ESM Table S2). This suggests a highly altered metabolism in the kidney tissue already 2 weeks after diabetes onset. The altered metabolites in the cortex and OS anatomical regions can be divided into six metabolite groups: non-esterified fatty acids (NEFAs); monoacylglycerols (MG); diacylglycerols (DG); short-chain and long-chain acylcarnitines; and amino acids. Species of NEFA, MG, DG, and short-chain and long-chain acylcarnitines had higher abundance in the diabetic kidney while amino acids had lower abundance in the diabetic tissue (ESM Table S5 and Fig. S1).

**Table 1:** Molecules detected using nano-DESI MSI analysis that have significantly altered signal intensity in rat kidney sections from STZ-treated rats (2 weeks post treatment) compared to control. C2, acetylcarnitine; C3, propionylcarnitine; C4, isobutyrylcarnitine; C4-OH, hydroxyisobutyrylcarnitine; C5, 2-methylbuturylcarnitine/isovalerylcarnitine; C16:0, palmitoylcarnitine; C18:2, linoleylcarnitine; C18:0, stearoylcarnitine. Mass error < 5 ppm

Increased signal in diabetes		Decreased signal in diabetes	
Chemical formula	Compound	Chemical formula	Compound
C_4_H_6_O_3_^b)^	2-Ketobutyric acid*	C_3_H_7_NO_3_	Serine ^b)^
C_6_H_8_O_4_^b)^	3-Hexenedioic acid*	C_2_H_7_NO_2_S	Hypotaurine ^b)^
C_6_H_10_O_5_^b)^	3-Hydroxymethylglutaric acid*	C_3_H_7_N_3_O_2_	Guanidinoacetic acid ^b)^
C_6_H_12_O_6_^b)^	Glucose*	C_5_H_9_NO_3_^b)^	Hydroxyproline*
C_9_H_17_NO_4_	C2 ^b)^	C_7_H_7_NO_2_^b)^	Anthranilic acid*
C_10_H_19_NO_4_	C3 ^b)^	C_7_H_13_NO_2_	Proline betaine ^b)^
C_11_H_21_NO_4_	C4 ^b)^	C_5_H_11_N_3_O_2_	4-Guanidinobutanoic acid ^b)^
C_12_H_23_NO_4_	C5 ^b)^	C_7_H_15_NO_2_^b)^	Dehydroxycarnitine*
C_11_H_21_NO_5_	C4-OH ^c)^	C_5_H_9_NO_4_	Glutamate ^a)^
C_18_H_30_O_2_	NEFA 18:3 ^b) d)^	C_6_H_9_N_3_O_2_	Histidine ^a)^
C_18_H_32_O_2_	NEFA 18:2 ^b) d)^	C_7_H_11_N_3_O_2_	Methylhistidine ^b)^
C_18_H_34_O_2_	NEFA 18:1 ^b) d)^	C_9_H_11_NO_3_	Tyrosine ^a)^
C_19_H_34_O_2_	Methyl linoleate ^b) d)^	C_11_H_12_N_2_O_2_	Tryptophan ^b)^
C_10_H_14_N_5_O_7_P	AMP ^b)^	C_38_H_76_NO_8_P	Phosphatidylcholine 30:0 ^b)^
C_21_H_36_O_4_	MG 18:3 ^b) d)^		
C_21_H_38_O_4_	MG 18:2 ^b) d)^		
C_21_H_40_O_4_	MG18:1 ^b) d)^		
C_23_H_45_NO_4_	C16:0 ^b)^		
C_25_H_45_NO_4_	C18:2 ^b)^		
C_25_H_49_NO_4_	C18:0 ^b)^		
C_35_H_68_O_5_	DG 32:0 ^d)^		
C_37_H_68_O_5_	DG 34:2 ^b) d)^		
C_37_H_70_O_5_	DG 34:1 ^b) d)^		
C_39_H_68_O_5_	DG 36:4 ^b) d)^		

*The most likely isomer

^a)^Level 1 identification through tandem mass spectrometry [22]

^b)^Level 2 identification through tandem mass spectrometry [22]

^c)^Level 3 identification through tandem mass spectrometry [22]

^d)^Presence or absence of double bond(s) confirmed by Ag^+^-adduct formation

### Specific lipid species accumulate in diabetic kidney tissue

**Fig. 3:**
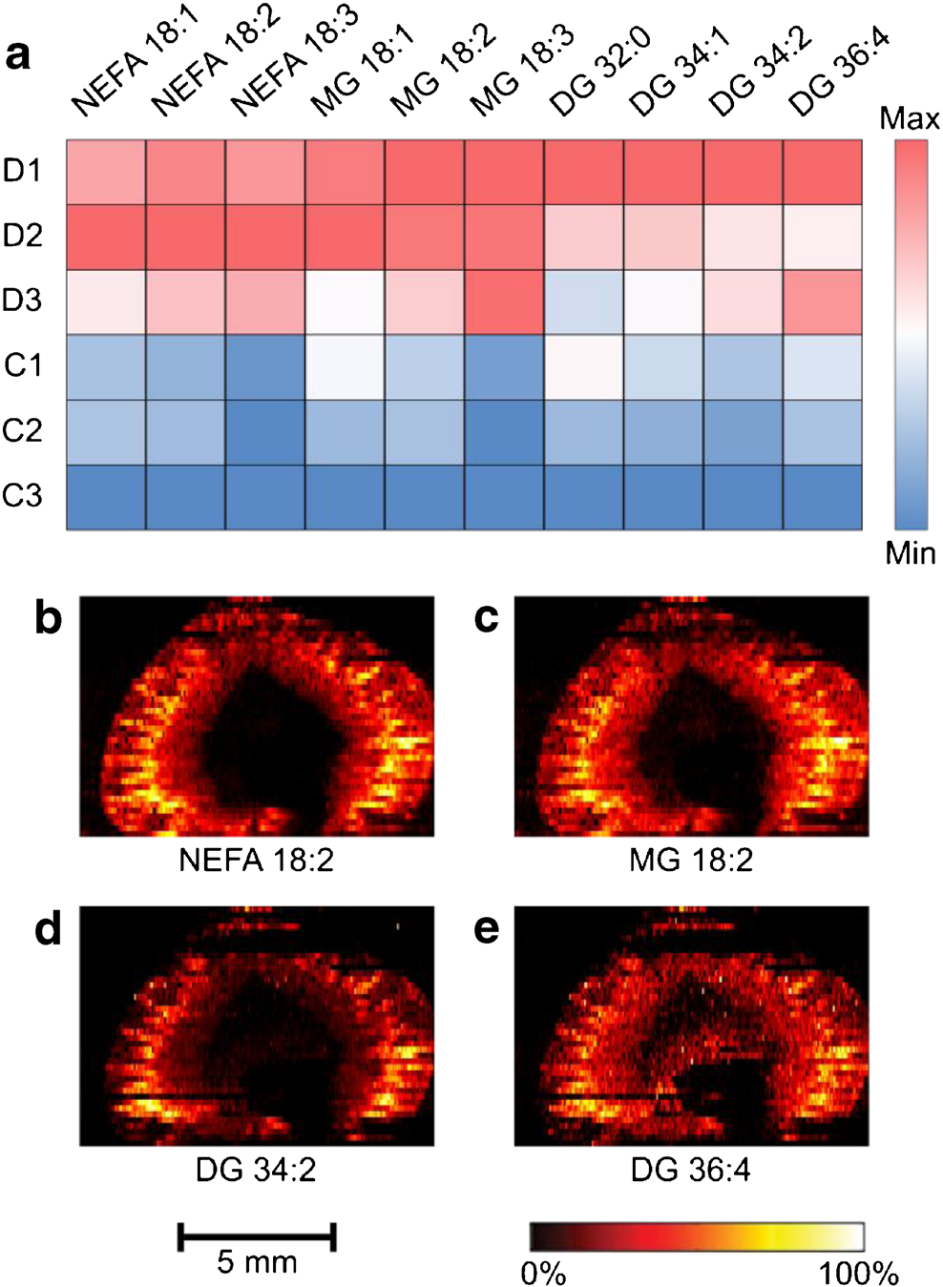
Untargeted nano-DESI MSI reveals increased NEFA, MG, and DG species in the cortex of diabetic kidney (*p* value < 0.05). (**a**) Heat map representing the mean signal intensities of three non-esterified fatty acids (NEFA), three monoacylglycerol (MG), and four diacylglycerol (DG) species. The rows represent diabetic rats (D1–D3) and control rats (C1–C3). The colour gradient spans from highest to lowest mean relative intensity across the 6 kidneys for each molecular species. Ion images of (**b**) [NEFA 18:2+K]^+^ (*m/z* 319.2035), (**c**) [MG 18:2+K]^+^ (*m/z* 393.2402), (**d**) [DG 34:2+K]^+^ (*m/z* 631.4699), and (**e**) [DG 36:4+K]^+^ (*m/z* 655.4699)

The altered lipid species NEFA, MG, and DG in the cortex and OS regions of diabetic kidney all contained 18 carbon unsaturated acyl chains. Specifically, the acyl chains of increased NEFA and MG species were 18:1, 18:2, and 18:3 (number of carbons:number of double bonds). In addition, the major constituents of the elevated DG species 32:0, 34:1, 34:2, and 36:4 also contained acyl chains 18:1, 18:2, and 18:3 (Fig. 3, ESM Table S7). These results suggest an important role of the metabolism for these moieties in DKD. However, while their abundances are significantly different (*p* value < 0.05) in the cortex and OS, their spatial distributions are conserved independent of diabetic state (ESM Fig. S2). In both control and STZ-treated tissue, the NEFA, MG, and DG species co-localise in the junction between the cortex and the medulla (OS, IS, and IM) with minor localisation to the IS and IM regions, suggesting the importance of this regional interface in DKD (Fig. 3b–d, ESM Fig. S3).

### Acylcarnitines accumulate in diabetic kidney tissue

**Fig. 4:**
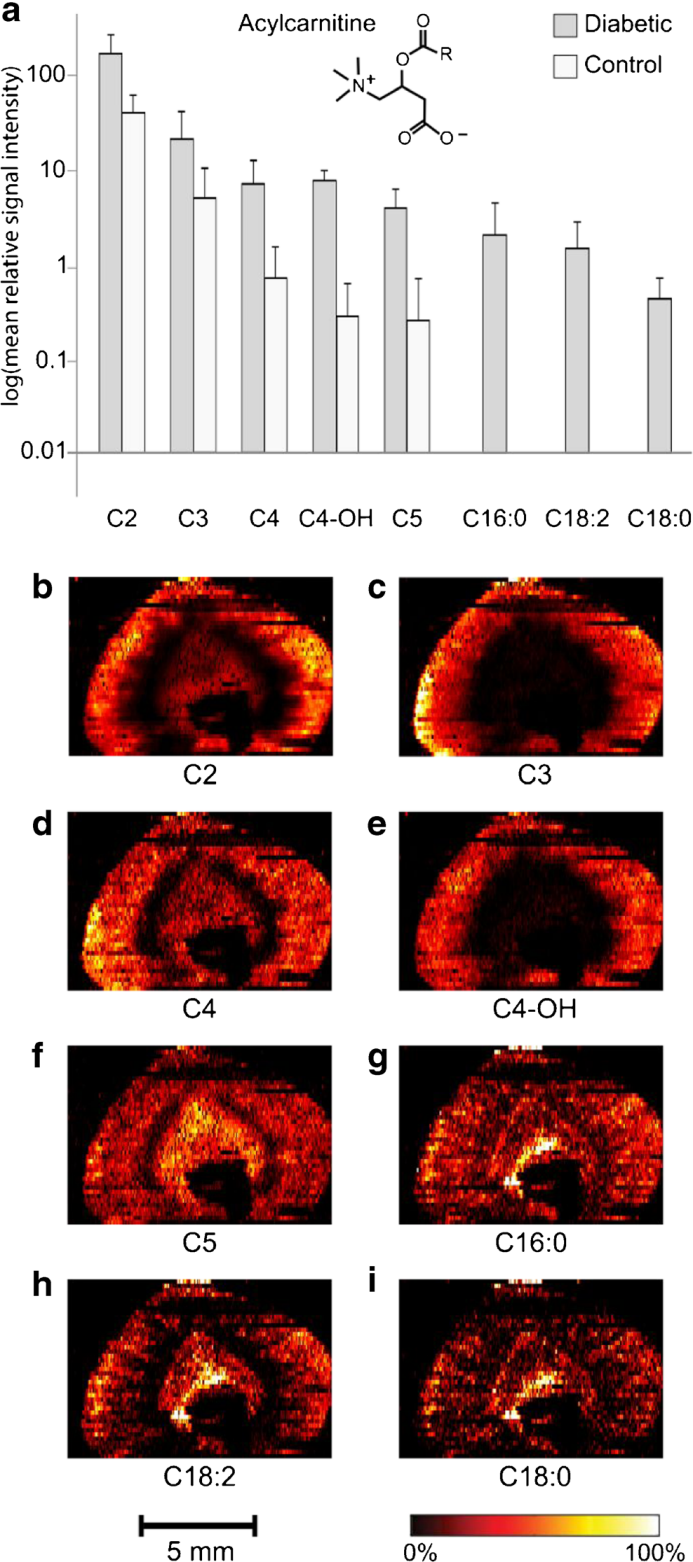
Accumulation of acylcarnitine species in the combined cortex and OS of diabetic kidney. (**a**) Graph displaying mean signal intensities of 8 acylcarnitine species in diabetic and control kidney on a logarithmic scale. Grey, diabetes; white, control. *n* = 3 each for diabetes and control. Error bars represent + 1 standard deviation. Ion images display (**b**) [C2+H]^+^ (*m/z* 204.1228), (**c**) [C3+H]^+^ (*m/z* 218.1386), (**d**) [C4+H]^+^ (*m/z* 232.1540), (**e**) [C4-OH+H]^+^(*m/z* 248.1490), (**f**) [C5+H]^+^ (*m/z* 246.1697), (**g**) [C16:0+H]^+^ (*m/z* 400.3422), (**h**) [C18:2+H]^+^ (*m/z* 424.3422), and (**i**) [C18:0+H]^+^ (*m/z* 428.3735); *p* < 0.01 except for C3 where *p* < 0.05

Both short- and long-chain acylcarnitine species were found significantly increased (*p* value < 0.01) in the cortex of diabetic kidney tissue compared to control. The large differences in signal intensities of the short-chain and long-chain acylcarnitines are displayed on a logarithmic scale in Fig. 4a, clearly showing the accumulation of acylcarnitines in the diabetic kidney. While the data in Fig. 4a is specific to the cortex and OS, the ion images in Fig. 4b–i show the distribution in the entire tissue section. The ion images reveal that the distribution of short-chain acylcarnitines is highly dependent on the acyl chain of the respective species (Fig. 4b–f). Specifically, all short-chain acylcarnitines are detected with high intensity in the cortex but show variable localisation to the OS and the medulla. While the localisation of C3 and isohydroxybutyrylcarnitine (C4-OH) to the medulla is minor (Fig. 4c, e), acetylcarnitine (C2), isobutyrylcarnitine (C4), and isovaleryl carnitine (C5) show some localisation in medullary regions (Fig. 4b, d, f). In contrast, all elevated long-chain acylcarnitines show more dispersed localisation across the diabetic kidney tissue section (Fig. 4g–i and ESM Fig. S4) and an almost complementary distribution to the altered NEFA, MG, and DG species that primarily localise to the OS of the kidney (Fig. 3b–e).

## Discussion

The localisation of individual metabolites to anatomical regions of the kidney suggests their respective importance for cellular and biological function in these regions. For example, the wide distribution of sphingomyelin in the tissue displays its general importance as a membrane lipid in the plasma membrane. Other localisations are, however, less known. For example, the distribution of methylhistidine and C3 to either side of the cortex-OS border is not known, but the minimal overlap of the respective ion images indicates that metabolic activities within these regions are different. Further, the medullary region is known to have a high salt concentration, and the high abundance of the osmoprotective compounds betaine and sorbitol evidences their importance in protecting cells from osmotic stress in this region [23]. Thus, the numerous metabolites detected by MSI and their abundances to cellular regions can be correlated to known biological activities.

The altered metabolite abundances in the kidney 2 weeks after STZ treatment show that despite no obvious histopathological changes, there are significant metabolic alterations within the tissue at this stage. An important region for DKD is the cortex as this is the site for blood filtration through the glomeruli. Further, a key part of disease progression is fibrosis of the tubuli, which occurs in the cortex. Here, we find that several lipid species accumulate to the cortex, which is in accordance with previous reports of renal lipid accumulation in response to hyperglycaemia [24–27]. However, the acyl chain composition of accumulating lipids and the pathophysiological role of the acyl chains 18:2 and 18:3 are not fully known. Overall, it is a common view that the mechanisms and possible toxic effects of renal lipid accumulation have been under-investigated [27–31]. In this context, it is of interest to note that the 18:2 and 18:3 acyl chains, which are the sole precursors of other NEFAs [32], are essential fatty acids that cannot be synthesised by mammals [33]. Further, dietary studies report that an increased intake of NEFA 18:2 and 18:3 is negatively associated with DKD, which supports their importance in disease development [34, 35]. One possible implication of the importance of NEFA 18:1, 18:2, and 18:3 and DG 18:2/18:2 is that they promote activation of protein kinase C (PKC) [36, 37]. PKC is highly involved in a number of hyperglycaemia-induced cellular responses and in DKD [38–40]. Thus, the accumulation of 18:1, 18:2, and 18:3 NEFA and DG species likely plays a key role in the progression of DKD.

The kidney has a high-energy demand for sustaining active transport, of small and large chemical substances throughout the nephron, which is further increased to maintain electrolyte and volume homeostasis following the elevated glomerular filtration rate in early diabetes. A common source for energy is the citric acid cycle; however, the kidney is mainly fuelled by the more ATP-efficient long-chain FAO in the mitochondrial matrix [41]. The rate-controlling step of FAO is esterification of fatty acids with carnitine, forming long-chain acylcarnitines [42]. This transformation is required for active transport of the fatty acids across the inner mitochondrial membrane. In addition to the elevated abundance of long-chain acylcarnitines, accumulation of the key component C2 further indicates a dysregulated FAO already at this early stage of diabetes. The final product of FAO is acetyl-CoA, which can be transesterified to C2 and either transported out of the kidney or used as a substrate in the citric acid cycle. C2 is the universal end product of mitochondrial metabolism; its increased abundance is therefore indicative of a perturbance of both the FAO and the citric acid cycle. Another major contributor of C2 is BCAA catabolism, and many of the short-chain acylcarnitines (C4, C4-OH, and C5) found increased in the kidney 2 weeks after disease onset are carnitine esters of key intermediates in the catabolism of leucine, isoleucine, and valine [43]. The BCAA catabolism product, C3, is converted to succinyl-CoA, another substrate of the citric acid cycle. Our results of apparent increases in both intermediates and products from catabolism of all three BCAAs in conjunction with decreases in several amino acids (AA) suggest an overall increase in AA metabolism. It is worth noting that the accumulation of C2 in diabetic kidney could be a result of either increased formation or reduced utilisation in the tissue. Regardless, the combined findings show a significant increase in substrates for the citric acid cycle, a requirement to meet the energy demands of hyperglycaemia.

## Conclusion

In this study, we provide new insights into local alterations of renal metabolism in early-stage insulinopenic diabetes. The results show significant lipid and acylcarnitine accumulation, indicating a dysregulation of the citric acid cycle, FAO, and BCAA metabolism in the cortex of diabetic kidney already 2 weeks after disease onset and before distinguishable histopathological changes. These results indicate that alterations in mitochondrial energy metabolism are associated with tubular and glomerular damage as a result of hyperglycaemia and insulin deficiency. Having demonstrated the suitability of nano-DESI MSI for detecting localised metabolic changes in kidney tissue, future studies should monitor the identified metabolite classes through the progression of diabetes and kidney disease. Comparative studies on different models of diabetes will also provide a better understanding of the origins and consequences of these metabolic alterations.

## Electronic supplementary material

ESM 1 (PDF 1226 kb)
