# Supplementary material for: Metabolite aberrations in early diabetes detected in rat kidney using mass spectrometry imaging
Source: Anal Bioanal Chem. 2019 Mar 20;411(13):2809–16. doi: 10.1007/s00216-019-01721-5 (PMC6522648; doi:10.1007/s00216-019-01721-5)
Supplement: Supplementary file 1 — (PDF 1226 kb) [file 216_2019_1721_MOESM1_ESM.pdf]

Analytical and Bioanalytical Chemistry

Electronic Supplementary Material

Metabolite aberrations in early diabetes detected in rat kidney using mass spectrometry imaging

Hilde-Marléne Bergman, Lina Lindfors, Fredrik Palm, Jan Kihlberg, Ingela Lanekoff

Tissue staining

The glass slide-mounted tissue sections were stained by sequential submersion in the following solutions: Xylene, 100%, 95%, 80% and 70% ethanol, H_2O, Mayers HTX, H_2O, eosin in 70% ethanol, 95% and 100% ethanol followed by mounting with Pertex (Histolab Products AB, Gothenburg, Sweden) in xylene.

Analyte identification

Significantly altered metabolites were tentatively assigned using Metlin and HMDB databases. For annotation, a selected ion monitoring (SIM) scan with a mass resolution of 140 000 ($m/\Delta m$ at m/z 200) was performed over a mass isolation window of 1.5 amu for the endogenous analyte of interest. Higher energy collisional dissociation was then used to generate a tandem mass spectrometry (MS/MS) spectrum by ramping the nominal collision energy from 10 to 80 while scanning between m/z 50 and the m/z of the precursor ion. Species containing double bonds were confirmed by silver adduct formation. Analysis was performed using a nano-DESI solvent consisting of 9:1 methanol:water (v:v) and 4 ppm silver ($AgNO_3$) and mass spectra were collected at m/z 200-1000 in positive mode at a resolution of 280 000 ($m/\Delta m$ at m/z 200).[1, 2]

Table S1 Physiological data of the insulinopenic diabetes rat model. Data presented as mean±SEM. Statistics calculated using unpaired Student's t-test

	**Control (*n*=4)**	**Diabetes (*n*=4)**	***p***
Body weight (g)	417±18	375±16	0.13
Blood glucose (mmol/l)	5.3±0.4	20.4±1.3	<0.0001
Left kidney weight (g)	1.46±0.09	2.05±0.09	0.004
Right kidney weight (g)	1.44±0.06	2.18±0.19	0.01
Mean arterial blood pressure (mmHg)	114±5	130±2	0.025
Urine flow (µl/min/kidney)	1.8±0.3	20.0±2.9	0.001
Hematocrit (%)	41±1	44±2	0.23
Glomerular filtration rate (ml/min/kidney)	1.11±0.08	2.13±0.12	0.001
Urinary protein excretion (µg/min/kidney)	58±3	128±6	<0.0001

Table S2 Identified molecules detected with decreased signal intensity in rat kidney sections from STZ-treated rats (2 weeks post treatment) compared to control. Mass error <5 ppm. *p <0.05, ** p <0.01. [*]The most likely isomer

**Chemical formula**	**Compound**	***m/z* for [M+H]$^+$**	***m/z* for [M+Na]$^+$**	***m/z* for [M+K]$^+$**
**C3H7NO3**	Serine [b)]		128.0317**	144.0055**
**C2H7NO2S**	Hypotaurine [b)]		132.0089**	147.9827**
**C3H7N3O2**	Guanidinoacetic acid [b)]	118.0613**	140.0427**	156.0167**
**C5H9NO3 [b)]**	Hydroxyproline[*]		154.0476**	170.0212**
**C7H7NO2 [b)]**	Anthranilic acid[*]		160.0366**	176.0105**
**C7H13NO2**	Proline betaine [b)]		166.0835**	182.0576**
**C5H11N3O2**	4-Guanidinobutanoic acid [b)]	146.0921**	168.073**	184.0481**
**C7H15NO2 [b)]**	Dehydroxycarnitine[*]		168.0991**	184.0732**
**C5H9NO4**	Glutamate [a)]		170.0421**	186.0161**
**C6H9N3O2**	Histidine [a)]		178.0586**	194.0324**
**C7H11N3O2**	Methylhistidine [b)]	170.0921**	192.0742**	208.0481**
**C9H11NO3**	Tyrosine [a)]		204.0626**	220.0367**
**C11H12N2O2**	Tryptophan [b)]		227.0787**	243.0527**
**C38H76NO8P**	Phosphatidylcholine 30:0 [b)]		728.5200*	744.4942*

a) Level 1 identification through tandem mass spectrometry[3]

b) Level 2 identification through tandem mass spectrometry[3]

Table S3 Identified molecules detected with increased signal intensity in rat kidney sections from STZ-treated rats (2 weeks post treatment) compared to control. NEFA = non-esterified fatty acid, MG = monoacylglycerol, DG = diacylglycerol. Mass error <5 ppm. *p <0.05, ** p <0.01. [*]The most likely isomer

Chemical formula	Example of compound	*m/z* for $[M+H]^+$	*m/z* for $[M+Na]^+$	*m/z* for $[M+K]^+$
**C4H6O3** a)	2-ketobutyric acid*		125.0210**	140.9944*
**C6H8O4** a)	3-Hexenedioic acid*		167.0312**	183.0052*
**C6H10O5** a)	3-hydroxymethylglutaric acid*		185.0418*	201.0157*
**C6H12O6** a)	Glucose*		203.0523**	219.0264**
**C9H17NO4**	C2 a)	204.1228**	226.1047*	242.0787*
**C10H19NO4**	C3 a)	218.1385*	240.1204*	256.0944*
**C11H21NO4**	C4 a)	232.154**	254.1359**	270.1099**
**C12H23NO4**	C5 a)	246.1697**	268.1516**	284.1256**
**C11H21NO5**	C4-OH a)	248.1491**	270.131**	286.105**
**C18H30O2**	NEFA 18:3 a) b)		301.2138**	317.1878**
**C18H32O2**	NEFA18:2 a) b)		303.2294**	319.2034**
**C18H34O2**	NEFA 18:1 a) b)		305.2451*	321.2191**
**C19H34O2**	Methyl linoleate a) b)		317.2446*	333.2188*
**C10H14N5O7P**	AMP a)		370.0504**	386.0243**
**C21H36O4**	MG 18:3 a) b)		375.2506**	391.2246**
**C21H38O4**	MG 18:2 a) b)		377.2662**	393.2402**
**C21H40O4**	MG18:1 a) b)		379.2819**	395.2559*
**C23H45NO4**	C16:0 a)	400.3422**		
**C25H45NO4**	C18:2 a) b)	424.3422**		
**C25H49NO4**	C18:0 a) b)	428.3735**		
**C35H68O5**	DG 32:0 a)		591.4959*	607.4699*
**C37H68O5**	DG 34:2 a) b)		615.4959**	631.4699**
**C37H70O5**	DG 34:1 a) b)		617.5115**	633.4855**
**C39H68O5**	DG 36:4 a) b)		639.4959*	655.4699*

a) Level 1 identification through tandem mass spectrometry[3]

b) Level 2 identification through tandem mass spectrometry[3]

Table S4 Molecules detected with different signal intensity in rat kidney sections from STZ-treated rats (2 weeks post treatment) compared to control. **p* <0.05, ** *p* <0.01

Chemical formula	Example of compound	*m/z* for $[M+H]^+$	*m/z* for $[M+Na]^+$	*m/z* for $[M+K]^+$
		**Decreased signal in diabetes**		
**C5H8N2O2**	Dihydrothymine		151.0475**	167.0215*
**C7H8N2O2**	N-methyl-2-pyridone-5-carboxamide		175.0476**	191.0218**
**C4H6N4O3**	Allantoin		181.0332**	197.0069**
**C8H17NO2**	2-Aminooctanoic acid	160.1328**	182.1150**	198.0889**
**C7H10O7/C8H14O2S2**	Homocitric acid/Lipoic Acid		229.0315**	245.0054**
**C9H13N3O4**	Deoxycytidine		250.0795**	266.0532*
**C13H14N2O2S2**	Wasalexin B		317.0385**	333.0130*
		**Increased signal in diabetes**		
**C17H18O4**	Sativan		309.1087**	325.0827*

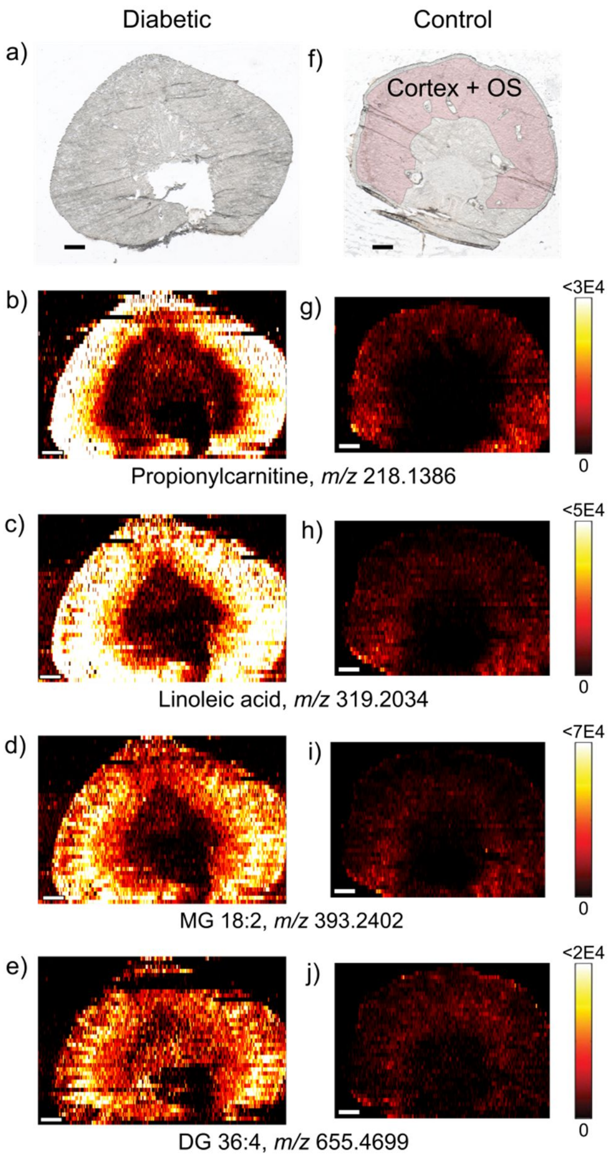

Fig. S1 Comparison of diabetic kidney and control kidney a) Optical image of diabetic kidney section. b) Ion image of [Propionylcarnitine+H]$^+$ in diabetic kidney, shown on an absolute scale ranging from 0 to <3E4. c) Ion image of [Linoleic acid+K]$^+$ in diabetic kidney, shown on an absolute scale ranging from 0 to <5E4. d) Ion image of [Monoacylglycerol 18:2+K]$^+$ in diabetic kidney, shown on an absolute scale ranging from 0 to <7E4 . e) Ion image of [Diacylglycerol 36:4+K]$^+$ in diabetic kidney, shown on an absolute scale ranging from 0 to <2E4 f) Optical image of control kidney section, with the region of interest highlighted. g) Ion image of [Propionylcarnitine+H]$^+$ in control kidney, shown on an absolute scale ranging from 0 to <3E4. h) Ion image of [Linoleic acid+K]$^+$ in control kidney, shown on an absolute scale ranging from 0 to <5E4. i) Ion image of [Monoacylglycerol 18:2+K]$^+$ in control kidney, shown on an absolute scale ranging from 0 to <7E4 . j) Ion image of [Diacylglycerol 36:4+K]$^+$ in control kidney, shown on an absolute scale ranging from 0 to <2E4. Scale bar = 1mm

Table S5 Mean relative intensities of selected differentially detected compounds in cortex+OS of rat kidney sections from STZ-treated rats (2 weeks post treatment) compared to control. $p<0.05$

**Compound**	**Diabetes (*n*=3)**	**Control (*n*=3)**
**NEFA 18:1**	6.86 ± 3.42	0.88 ± 0.61
**NEFA 18:2**	44.99 ± 8.56	9.37 ± 4.85
**NEFA 18:3**	2.47 ± 0.43	0.04 ± 0.07
**MG 18:1**	15.37 ± 7.89	3.16 ± 2.63
**MG 18:2**	40.45 ± 8.59	10.48 ± 6.41
**MG 18:3**	1.16 ± 0.02	0.04 ± 0.06
**DG 32:0**	7.81 ± 6.38	1.75 ± 1.31
**DG 34:1**	4.71 ± 3.77	0.43 ± 0.37
**DG 34:2**	12.87 ± 7.23	1.77 ± 1.35
**DG 36:4**	3.57 ± 1.45	0.84 ± 0.60
**C2**	172.87 ± 69.01	41.25 ± 6.58
**C3**	24.24 ± 11.00	6.26 ± 1.71
**C4**	8.25 ± 1.77	0.78 ± 0.18
**C4OH**	7.93 ± 3.83	0.30 ± 0.10
**C5**	4.05 ± 1.39	0.28 ± 0.17
**C16:0**	2.23 ± 2.22	-
**C18:2**	1.55 ± 1.08	-
**C18:0**	0.48 ± 0.48	-

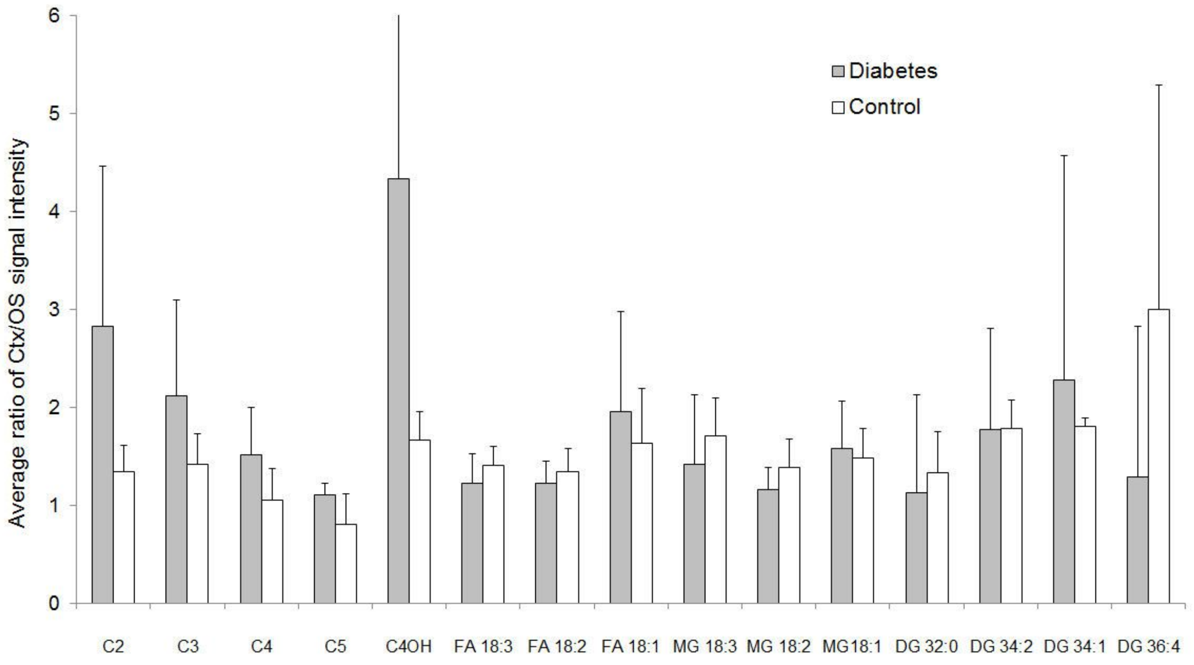

Fig. S2 The spatial distributions of compounds are conserved in diabetic and control kidney. Graph displaying mean ratios of cortex:OS signal intensities of 15 species in diabetic and control kidney. Grey = Diabetes, White = Control. n=3 each for diabetes and control. Error bars represent +1 standard deviation. Welch's t-test p-values range from 0.23 to 0.99

Table S6 Tandem mass spectrometry data of acylcarnitines detected in kidney, compared to fragmentation pattern of database values obtained from Metlin (https://metlin.scripps.edu/landing_page.php?pgcontent=mainPage) or the human metabolome database (HMDB, http://www.hmdb.ca/). The relative intensities are given at a normalized collision energy (NCE) of 20 to 40 for the endogenous acylcarnitines and at 10V for database values

**Compound**	**Measured *m/z***	**Theoretical *m/z***	**Detected fragment *m/z* (Relative intensity in %)**
**Acetylcarnitine$_{kidney}$ $[M+H]^+$**	204.1231	204.1230	204.1231 (100), 85.0290 (35), 145.0496 (16), 60.0816 (9)
**Acetylcarnitine$_{Metlin}$ $[M+H]^+$**	204.1223	204.1230	204.1223 (100), 85.0283 (53), 145.0506 (16), 60.0821 (14)
**Propionylcarnitine$_{kidney}$ $[M+H]^+$**	218.1385	218.1387	218.1385 (100), 85.0289 (60), 159.0651 (12)
**Propionylcarnitine$_{Metlin}$ $[M+H]^+$**	218.1380	218.1387	218.1380 (100), 85.0288 (73), 159.0642 (21), 60.0815 (12)
**Butyrylcarnitine$_{kidney}$/ Isobutyrylcarnitine$_{kidney}$ $[M+H]^+$**	232.1541	232.1543	232.1540 (100), 85.0289 (75), 173.0807 (22), 60.0815 (4)
**Butyrylcarnitine$_{Metlin}$ $[M+H]^+$**	232.1571	232.1543	232.1565 (100), 85.0295 (59), 173.0821 (20), 60.0818 (9)
**Isobutyrylcarnitine$_{Metlin}$ $[M+H]^+$**	232.1541	232.1543	232.1540 (100), 85.0291 (30), 173.0796 (21), 60.0818 (8)
**Isovalerylcarnitine$_{kidney}$/ 2-Methylbutyroylcarnitine$_{kidney}$ $[M+H]^+$**	246.1697	246.1700	246.1697 (100), 85.0290 (55), 187.0964 (12)
**2-Methylbutyroylcarnitine$_{Metlin}$ $[M+H]^+$**	246.1716	246.1700	246.1713 (100), 85.0295 (40), 187.0917 (18), 60.0815 (6)
**Hydroxybutyrylcarnitine$_{kidney}$ $[M+H]^+$**	248.1489	248.1493	85.0289 (100), 248.1489 (45), 189.0756 (2), 60.0815 (2)
**Hydroxybutyrylcarnitine$_{database}$**	-	-	-
**Palmiotylcarnitine$_{kidney}$ $[M+H]^+$**	400.3414	400.3424	400.3426 (100), 85.0291 (90), 60.0817 (14), 341.2689 (10), 144.1021 (2.5)
**Palmitoylcarnitine$_{data}$ $[M+H]^+$**	400.3413	400.3424	400.3416 (100), 85.0279 (73), 341.2674 (10), 60.0807 (9)
**Linoleyl carnitine$_{kidney}$ $[M+H]^+$**	424.3409	424.3421	424.3426 (100), 85.0291 (50), 60.0817 (8)
**Linoleyl carnitine$_{Metlin}$ (*in silico*), $[M+H]^+$**	-	424.3421	144.1020 (100), 85.0284 (31)
**Linoleyl carnitine$_{HMDB}$ (*in silico*), $[M+H]^+$**	-	424.3421	144.1020 (100), 85.0284 (12), 60.0808 (6)
**Stearoylcarnitine$_{kidney}$ $[M+H]^+$**	428.3725	428.3734	428.3739 (100), 309.2791 (70), 85.0291 (27), 60.0817 (4)
**Stearoylcarnitine$_{HMDB}$ $[M+H]^+$**	428	428.3734	**At 10V:** 427 (100), 428 (61), 85 (10) **At 20 V:** 85 (100), 60 (18)

Table S7 Tandem mass spectrometry data of fatty acids detected in kidney compared to fragmentation pattern of database values obtained from Metlin (https://metlin.scripps.edu/landing_page.php?pgcontent=mainPage) or the human metabolome database (HMDB, http://www.hmdb.ca/). NEFA = Non-esterified fatty acid. The relative intensities are given at a normalized collision energy (NCE) of 40 for the endogenous compounds and at 20V for database values unless otherwise stated

**Compound**	**Measured *m/z***	**Theoretical *m/z***	**Detected fragment *m/z* (Relative intensity in %)**
**NEFA 18:3$_{kidney}$ [M-H]$^-$**	277.2169	277.2173	277.2173 (100), 59.0121 (9), 259.2086 (3), 233.2271 (1)
**γ-Linolenic acid$_{Metlin}$ [M-H]$^-$**	277.2166	277.2173	**At 10 V** : 277.2167 (100), 209.1141 (5), 233.2282 (3), 59.0121 (3)
**α- Linolenic acid$_{Metlin}$ [M-H]$^-$**	277.2166	277.2173	**At 20 V:** 277.2151 (100), 59.0144 (6), 259.2047 (2)
**NEFA 18:2$_{kidney}$ [M-H]$^-$**	279.2330	279.2330	279.2329 (100), 261.2227 (2), 59.0121 (2)
**Linoleic acid$_{Metlin}$ [M-H]$^-$**	279.2330	279.2330	279.2330 (100), 261.0024 (1), 59.0138 (1)
**NEFA 18:1$_{kidney}$ [M-H]$^-$**	281.2476	281.2486	281.2482 (100), 59.0121 (0.35), 263.2382 (0.12)
**Oleic acid $_{Metlin}$ [M-H]$^-$**	281.2497	281.2486	281.2492 (100)
**Oleic acid$_{HMDB}$ (*In silico*), [M-H]$^-$**	-	281.2486	281.2481 (100), 237.2582 (52), 263.2375 (13), 59.0133 (15)

Table S8 Tandem mass spectrometry data of monoacylglycerols detected in kidney compared to fragmentation pattern of database values obtained from Metlin (https://metlin.scripps.edu/landing_page.php?pgcontent=mainPage).). MG = monoacylglycerol, DG = diacylglycerol The relative intensities are given at a normalized collision energy (NCE) of 10 for the endogenous compounds unless otherwise stated and at 10V for database values

Compound	Measured *m/z*	Theoretical *m/z*	Detected fragment *m/z* (Relative intensity in %)
**MG 18:3$_{kidney}$ [M+H]$^+$**	353.2665	353.2686	**At NCE 20:** 353.2663 (100), 261.2215 (2)
**MG 18:3$_{Metlin}$** *In silico*, [M+H]$^+$	-	353.2686	353.269 (100), 261.221 (45), 335.258 (45)
**MG 18:2$_{kidney}$ [M+H]$^+$**	355.2838	355.2843	263.2372 (100), 245.2266 (65), 337.2741 (50), 281.2475 (10), 81.0706 (2),95.0862 (2.5) 109.1017 (4), 123.1172 (5)
**MG 18:2$_{Metlin}$ [M+H]$^+$**	355.2887	355.2843	263.2363 (100), 245.2258 (77), 337.2730 (58), 81.0702 (18), 95.0854 (16), 109.1007 (15), 123.1160 (11) 281.2466 (2)
**MG 18:1$_{kidney}$ [M+H]$^+$**	357.2300	357.2999	265.2527 (100), 339.2896 (75), 247.2422(35), 283.2633 (20), 121.1016 (6.5), 135.1171 (6.5), 149.1327 (4), 109.1017 (3.5), 123.1172 (3.5), 95.0861 (3.5), 165.1640 (3.5), 151.1483 (3), 81.0706 (2.5), 163.1483 (2.5), 97.0108 (2), 83.0862 (1.5)
**MG 18:1$_{Metlin}$ [M+H]$^+$**	357.2989	357.2999	265.2517 (100), 339.2898 (88), 247.2415 (45), 121.1014 (18), 135.1166 (16), 95.0852(14), 81.0703 (12), 283.2643 (12), 83.0860 (11), 109.1009 (12), 97.1015 (10), 149.1316 (10), 165.1632 (5), 163.1482 (5), 123.1164 (4) 151.1478 (3),
**DG 32:0$_{kidney}$ [M+H]$^+$**	569.5140	569.5140	313.2740 (100), 551.5045 (75), 341.3054 (0.5), 285.2428 (0.1)
**DG (16:0/16:0)$_{Metlin}$** *In silico* [M+H]$^+$	569.5140	569.5140	569.5140 (100), 239.2370 (44), 551.5030 (27) 313.2740 (19)
**DG (18:0/14:0)$_{Metlin}$** *In silico*, [M+H]$^+$	569.5140	569.5140	569.5140 (100), 341.3050 (87), 285.2420 (25), 551.5030 (6)
**DG 34:1$_{kidney}$ [M+H]$^+$**	595.5308	595.5296	313.2739 (100), 577.5204 (85), 339.2896 (70), 341.3053 (1), 311.2582 (0.4)
**DG 18:1/16:0$_{Metlin}$** *In silico*, [M+H]$^+$	-	595.5296	595.5300 (100), 313.2740 (81) 339.2890 (25), 577.519 (6)
**DG 18:0/16:1$_{Metlin}$** *In silico*, [M+H]$^+$	-	595.5296	595.5300 (100), 341.3050 (81), 311.2580 (25), 577.519 (12)
**DG 34:2$_{kidney}$ [M+H]$^+$**	593.5139	593.5140	313.2740 (100), 575.5046 (55), 337.2740 (50), 311.5284 (0.4), 339.2896 (0.6)
**DG (16:0/18:2)$_{Metlin}$** *In silico*, [M+H]$^+$	-	593.5140	593.5140 (100), 313.2740 (80), 337.2740 (26), 575.5030 (26)
**DG (18:1/16:1)$_{Metlin}$** *In silico*, [M+H]$^+$	-	593.5140	593.5140 (100), 339.2890 (86), 311.2580 (26), 575.5030 (20)
**DG 36:4$_{kidney}$ [M+H]$^+$**	617.5124	617.5140	617.5121 (100), 599.5046 (3), 337.2739 (2.5), 313.2739, (1), 339.2895 (0.7), 335.2583 (0.6), 263.2370 (0.15), 361.2739 (0.15)
**DG (18:2/18:2)$_{Metlin}$** *In silico*, [M+H]$^+$	-	617.5140	337.2740 (100), 617.5140 (85), 263.2370 (60), 355.2840 (40), 599.5030 (30)
**DG (16:0/20:4)$_{Metlin}$** *In silico*, [M+H]$^+$	-	617.5140	617.5140 (100), 313.2740 (80), 599.5030 (40), 361.2740 (26)
**DG (18:1/18:3)$_{Metlin}$** *In silico*, [M+H]$^+$	-	617.5140	617.5140 (100), 339.2890 (88), 335.2580 (33), 599.5030 (16)

Table S9 Tandem mass spectrometry data of compounds detected with increased signal intensity in diabetic kidney as compared to control. The fragmentation pattern of the analyte has been compared to database values obtained from Metlin (https://metlin.scripps.edu/landing_page.php?pgcontent=mainPage), The relative intensities are given at the specified normalized collision energy (NCE) and voltage

**Compound**	**Measured *m/z***	**Theoretical *m/z***	**Detected fragment *m/z* (Relative intensity in %)**
**C4H6O3** **[M+H]$^+$**	103.0395	103.0390	**At NCE 20:** 85.0289 (700), 103.0395 (650), 59.0599 (350), 57.0343 (60),
**E.g. 2-ketobutyric acid$_{HMDB}$** ***In silico,*** [M+H]$^+$		103.0390	**At 10 V:** 85.0290 (100), 103.0395 (96), 57.0340 (53), 67.0184 (24), 55.0184 (5), 59.0497 (4)
**C6H8O4** **[M+H]$^+$**	145.0495	145.0495	**At NCE 20:** 127.0391 (100), 109.0288 (78), 99.0445 (63), 145.0495 (38)
**E.g. 3-hexenedioic acid $_{Metlin}$** ***In silico,*** [M+H]$^+$		145.0495	**At 10 V**: 145.0500 (100), 127.0390 (76), 99.0441 (41), 109.0280 (11)
**C6H10O5** **[M+H]$^+$**	163.0600	163.0601	**At NCE 10:** 85.0290 (14 000), 103.0394 (25), 99.0445 (21), 127.0391 (14), 163.0603 (350),
**3-hydroxy-3-methyl-Glutaric acid$_{Metlin}$** **[M+H]$^+$**	163.0604	163.0601	**At 10 V**: 103.0388 (100), 127.0385 (91), 85.0284 (58), 99.0428 (50)
**C6H12O6** **[M+Na]$^+$**		203.0526	**At NCE 10:** 203.0524 (100) **At NCE 80:** 72.0451 (100) , 55.0551 (19), 129.0700 (21), 98.0969 (12), 119.0857 (8), 131.0492 (7), 95.0860 (6), 203.0526 (6), 86.0970 (2),
**E.g. Myo-inositol$_{Metlin}$** **[M+Na]$^+$**	203.0522	203.0526	**At 10V**: 203.0518 (100) **At 20V**: 203.0480 (100), 72.0469 (32), 98.0976 (33), 100.1123 (30), 95.0843 (20), 131.0467 (18), 59.0455 (16), 119.0841 (16), 90.7844 (17), 142.1134 (16), 70.0644 (15), 129.0644 (15) **At 40V:** (44.0500 (100)), 55.0559 (65), 73.0533 (44), 86.0995 (43), 156.0758 (28)
**Methyl linoleate** **[M+H]$^+$**	295.2623	295.2632	**At NCE 10:** 263.2368 (3 300), 245.2262 (91), 295.2630 (15) **At NCE 40:** 81.0705 (2000), 69.0706 (90), 95.0860 (90), 67.0550 (75), 83.0861 (55), 97.1017 (40), 109.1015 (40), 121.1014 (28), 133.1013 (28), 55.0551 (15), 163.1481 (6)
**Methyl linoleate$_{Metlin}$** **[M+H]$^+$**	295.2649	295.2632	**At NCE 10:** 263.2393 (100), 245.2249 (80), 295.2649 (25), 69.0712 (28), 109.1007 (23), 121.1003 (20), 133.1020 (20)163.1472 (20), 83.0861 (19), 81.0708 (18), 95.0882 (18), 97.1024 (17), 55.0561 (15), 67.0541 (5),
**AMP** **[M+H]$^+$**	348.0703	348.0704	**At NCE 10:** 348.0702 (100), 136.0618 (90) **At NCE 80:** 136.0618 (100), 119.0355 (14), 97.0289 (13)
**AMP$_{Metlin}$** **[M+H]$^+$**	348.0689	348.0704	**At 10 V:** 348.0689 (100), 136.0631 (90) **At 40V:** 136.0615 (100), 97.0298 (13), 119.0322 (8)

Table S10 Tandem mass spectrometry data of compounds detected with decreased signal intensity in diabetic kidney as compared to control. The fragmentation pattern of the analyte has been compared to database values obtained from Metlin (https://metlin.scripps.edu/landing_page.php?pgcontent=mainPage), the human metabolome database (HMDB, http://www.hmdb.ca/) or the pattern of an analytical standard (std). The relative intensities are given at a normalized collision energy (NCE) of 10 for experimental data and at 10V for database values unless otherwise stated. For the detected compounds with a nominal mass of 146, the fragment with a mass ~87.0450 amu was shared between different chemical formulas (highlighted with bold text)

**Compound**	**Measured *m/z***	**Theoretical *m/z***	**Detected fragment *m/z* (Relative intensity in %)**
**Serine** **$[M+H]^+$**	106.0502	106.0499	60.0452 (100), 88.0399 (15), 106.0503 (5), 70.0294 (1.5)
**Serine$_{Metlin}$** **$[M+H]^+$**	106.0496	106.0499	60.0454 (100), 64.0164 (15), 88.0398 (8), 106.0496 (8), 70.0294 (5)
**Hypotaurine** **$[M+H]^+$**	110.0274	110.0270	92.0170 (100), 110.0274 (91)
**Hypotaurine$_{Metlin}$** **$[M+H]^+$**	110.0270	110.0270	110.0270 (100), 92.0166 (71), 64.9698 (33)
**Guanidinoacetic acid** **$[M+H]^+$**	118.0617	118.0611	76.0397 (100), 72.0561 (81), 101.0348 (68), 118.0617 (36), 73.0401 (18)
**Guanidinoacetic acid$_{Metlin}$** **$[M+H]^+$**	118.0609	118.0611	118.0609 (100) 72.0562 (75), 76.0396 (59), 101.0345 (37), 55.0297 (27), 73.0404 (27)
**C5H9NO3** **$[M+H]^+$**	132.0659	132.0655	132.0659 (7 000), 86.0606 (14 000) 114.0553 (350), 68.0502 (2 500), 72.0451 (150), 73.0291 (25)
**E.g. 4-Hydroxyproline$_{Metlin}$** **$[M+H]^+$**	132.0660	132.0655	114.0550 (100), 132.0660 (81), 86.0600 (4), 68.0495 (2), 96.0444 (2)
**E.g. 5-Aminolevulinic acid$_{Metlin}$** **$[M+H]^+$**	132.0645	132.0655	86.0600 (100), 114.0543 (98) 132.0645 (20), 55.0184 (12), 68.0495 (8) 53.0391 (4), 72.0445 (3), 73.0284 (3)
**C7H7NO2** **$[M+H]^+$**	138.0549	138.0550	**At NCE 80:** 94.0656 (100), 138.0550 (90), 65.0386 (10), 92.0500 (3), 93.0579 (5), 121.0286 (3), 120.0446 (0.16)
**E.g. Aminobenzoic acid$_{Metlin}$** **$[M+H]^+$**	138.0549	138.0550	At 20 V: 77.0394 (100), 94.0655 (50), 65.0394 (50), 120.0433 (35), 138.0542 (33), 92.0493 (27), 93.0572 (24), 121.0280 (6)
**Proline betaine** **$[M+H]^+$**	144.1019	144.1019	**At NCE 80:** 144.1019 (100), 58.0659 (90), 84.0814 (75)
**Proline betaine[a)]** **$[M+H]^+$**	144	144.1019	144 → 58<br>144 → 84
**Guanidinobutanoic acid** **$[M+H]^+$**	146.0925	146.0924	146.0924 (100), **87.0446** (83), 86.0606 (25), 104.0710 (14), 60.0564 (8), 128.0820 (8), 111.0557 (3)
**Guanidinobutanoic acid$_{Metlin}$** **$[M+H]^+$**		146.0924	146.0917 (100**), 87.0447** (35), 86.0607 (32), 60.0565 (9), 104.0707 (8), 128.0817(6) 111.0553 (4), 69.0342 (4)

**C7H15NO2**<br>**[M+H]$^+$**	146.1176	146.1176	**87.0441** (100), 146.1175 (78), 60.0816 (19), 100.1126 (3)
**E.g. Acetylcholine$_{Metlin}$**<br>**[M+H]$^+$**	146.1177	146.1176	**87.0448** (100), 146.1177 (29), 60.0818 (6), 64.0165 (5)
**E.g. Dehydroxycarnitine$_{Metlin}$**<br>*In silico,* [M+H]$^+$	146.1180	146.1176	146.1180 (100), 128.1070 (67), 100.1120 (20), 98.0964 (12), 102.1280 (7), 110.0960 (5), **87.0441 (5)**
**Glutamate**<br>**[M+H]$^+$**	148.0604	148.0604	84.0450 (100), 102.0554 (50), 130.0500 (40), 148.0604 (14)
**Glutamate$_{Std}$**<br>**[M+H]$^+$**	148.0603	148.0604	84.0449 (100), 130.0499 (45), 102.0553 (40), 148.0603 (12)
**Histidine**<br>**[M+H]$^+$**	156.0767	156.0768	110.0716 (100), 156.0767 (33), 95.0609 (5), 83.0610 (1), 93.0453 (1)
**Histidine$_{Std}$**<br>**[M+H]$^+$**	156.0767	156.0768	110.0715 (100), 156.0766 (30), 95.0608 (6), 83.0609 (2), 93.0452 (1.5), 138.1100 (1)
**Methylhistidine**<br>**[M+H]$^+$**	170.0925	170.0924	170.0925 (100), 124.0872 (50), 109.0764 (20), 126.1028 (20), 96.0687 (8), 97.0765 (5), 127.0712 (5), 153.0659 (2)
**1-Methylhistidine$_{Metlin}$**<br>***In silico***, **[M+H]$^+$**	-	170.0924	124.0870 (100), 170.0920 (84), 153.0660 (17), 107.0600 (10), 152.0820 (10)
**3-Methylhistidine$_{Metlin}$**<br>**[M+H]$^+$**	170.0923	170.0924	170.0923 (100), 96.0684 (49), 109.0761 (9), 126.1023 (14), 95.0606 (10), 97.0761 (9), 125.0707 (3)
**Tyrosine**<br>**[M+H]$^+$**	182.0812	182.0812	136.0758 (100), 165.0547 (75), 123.0443 (20), 147.0441 (13), 119.0494 (9), 182.0812 (12)
**Tyrosine$_{Std}$**<br>**[M+H]$^+$**	182.0810	182.0812	136.0756 (100), 165.0545 (70), 123.0441 (25), 119.0493 (14), 147.0439 (23), 129.0556 (16), 137.0894 (12), 182.0810 (8), 91.0547 (2)
**Tryptophan**<br>**[M+H]$^+$**	205.0968	205.0972	188.0707 (80 000), 146.0601 (13), 205.0972 (0.25)
**Tryptophan $_{Metlin}$**<br>**[M+H]$^+$**	205.0966	205.0972	188.0704 (100), 146.0598 (20), 205.0975 (3)
**Phosphatidylcholine 30:0**<br>**[M+H]$^+$**	706.5389	706.5381	At NCE 20: 184.0734 (100), (20), 706.5389 (7), 86.0970 (1), 104.1075 (1), 450.2982 (0.1), 468.3088 (0.15), 478.3294 (0.1), 496.3403 (0.1), 523.4727 (0.05)
**PC 16:0/14:0$_{HMDB}$**<br>***In silico***, **[M+H]$^+$**	-	706.5381	184.0739 (100), 86.0970 (17), 104.1075 (17), 125.0004 (17), 450.2984 (17), 468.3090 (17), 478.3297 (17), 496.3403 (17), 523.4726 (17), 647.4652 (17), 688.5281 (17), 706.5387 (17)

a) Ref[4]

Table S11 Mass spectrometry data of endogenous substances in kidney with silver added to the nano-DESI solvent. NEFA = non-esterified fatty acid, MG = monoacylglycerol, DG = diacylglycerol

Compound	Theoretical *m/z* [M+ ^{107}Ag]$^+$	Measured *m/z*	Theoretical *m/z* [M+ ^{109}Ag]$^+$	Measured *m/z*	Ratio 107/109 (theoretically 1.08)
**NEFA 18:3**	387.1447	387.1449	389.1444	389.1445	1.10
**NEFA 18:2**	385.1291	385.1295	387.1288	387.1296	1.33
**NEFA 18:1**	389.1604	389.1599	391.1601	391.1600	1.03
**Methyl linoleate**	401.1610	401.1606	403.1607	403.1604	1.07
**MG 18:3**	459.1659	459.1663	461.1656	461.1662	1.16
**MG 18:2**	461.1815	461.1818	463.1812	463.1816	1.10
**MG 18:1**	463.1972	463.1973	465.1969	465.1972	1.08
**DG 32:0**	675.4112	n.d.	677.4109	n.d.	-
**DG 34:1**	699.4112	699.4118	701.4109	701.4116	1.10
**DG 34:2**	701.4268	701.4271	703.4265	703.4271	1.40
**DG 36:4**	723.4112	723.4118	725.4109	423.4116	1.13

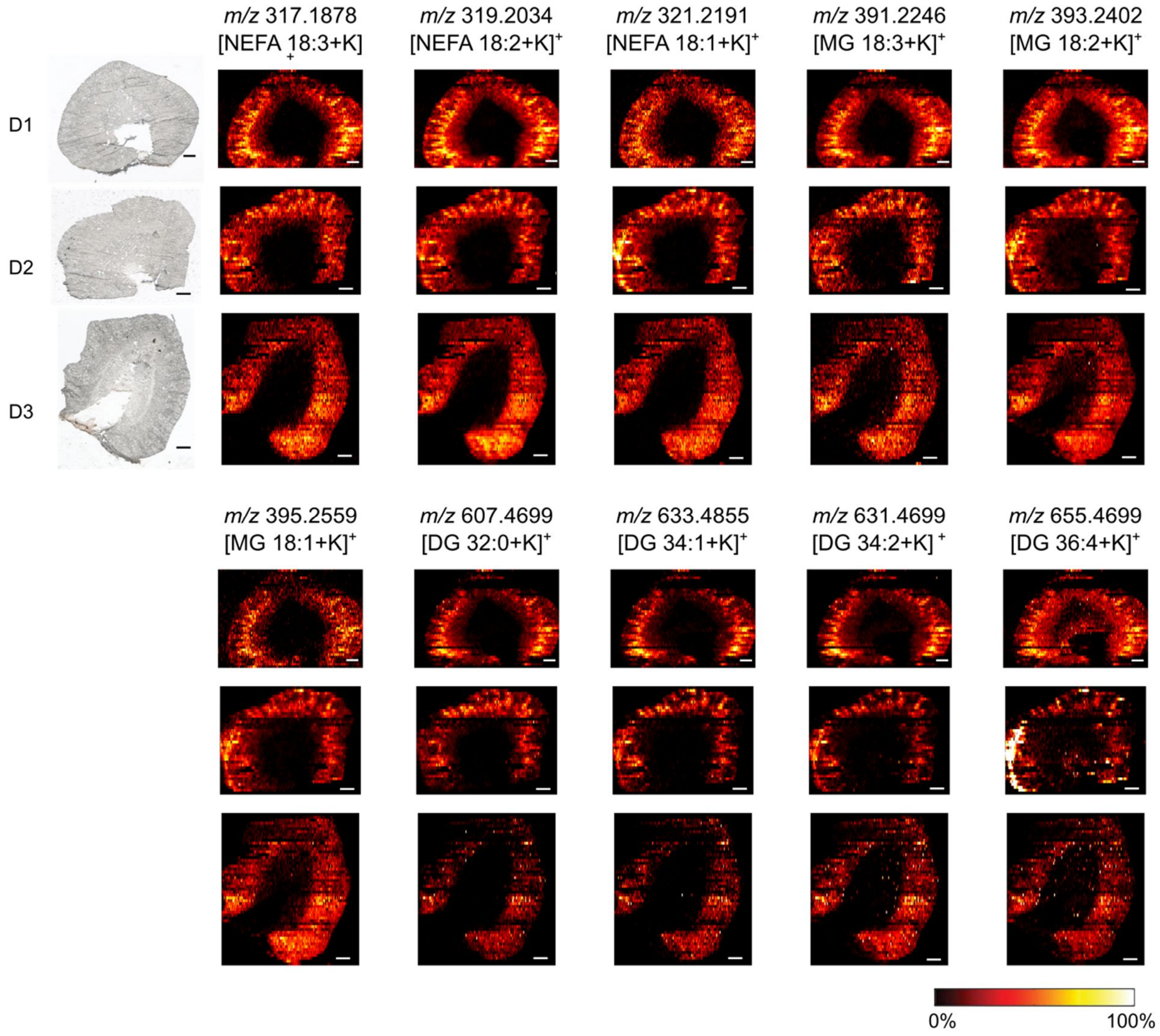

Fig. S3 Ion images of fatty acid- and neutral lipid species in kidney tissue from three diabetic rats 2 weeks post STZ-treatment. The signal intensity is given on a relative scale ranging from dark to bright. Scale bar = 1 mm. NEFA = non-esterified fatty acid, MG = monoacylglycerol, DG = diacylglycerol

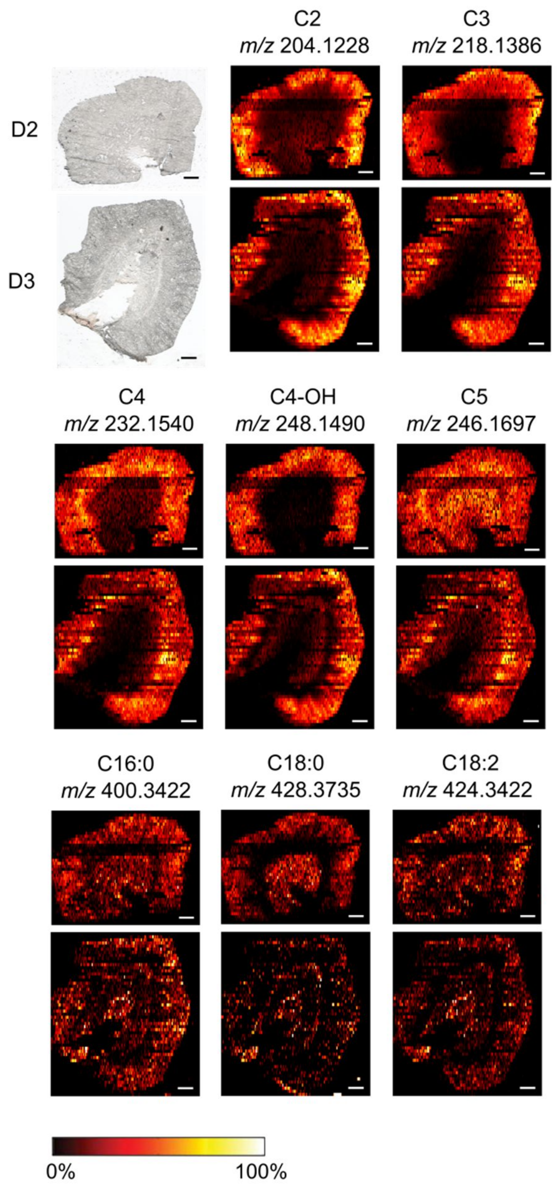

Fig. S4 Ion images of acylcarnitine species in kidney tissue from two diabetic rats 2 weeks post STZ-treatment. Acylcarnitine images for diabetic rat 1 can be found in the main paper. The signal intensity is given on a relative scale ranging from dark to bright. Scale bar = 1 mm

References

[1] Duncan KD, Fang R, Yuan J, et al. (2018) Quantitative Mass Spectrometry Imaging of Prostaglandins as Silver Ion Adducts with Nanospray Desorption Electrospray Ionization. Anal Chem. 90: 7246-7252
[2] Frenking G, Fröhlich N (2000) The Nature of the Bonding in Transition-Metal Compounds. Chem Rev 100: 717-774
[3] Sumner LW, Amberg A, Barrett D, et al. (2007) Proposed minimum reporting standards for chemical analysis Chemical Analysis Working Group (CAWG) Metabolomics Standards Initiative (MSI). Metabolomics : Official journal of the Metabolomic Society 3: 211-221
[4] Lang R, Lang T, Bader M, Beusch A, Schlagbauer V, Hofmann T (2017) High-Throughput Quantitation of Proline Betaine in Foods and Suitability as a Valid Biomarker for Citrus Consumption. J Agric Food Chem 65: 1613-1619
